# Analysis of interhemispheric EEG spectral variation at rest and after mental arithmetic task in schizophrenic patients compared to healthy controls

**DOI:** 10.1192/j.eurpsy.2025.2114

**Published:** 2025-08-26

**Authors:** N. Smaoui, D. Jardak, R. Feki, S. Omri, I. Gassara, N. Charfi, J. Ben Thabet, M. Maalej, M. Bou Ali Maalej, L. Zouari, L. Triki

**Affiliations:** 1 Psychiatry C, Hedi Chaker University Hospital; 2 Functional Explorations, Habib Bourguiba University Hospital, Sfax, Tunisia

## Abstract

**Introduction:**

Electroencephalogram (EEG) has emerged to be valuable for understanding the neurophysiological mechanisms underlying cognitive dysfunctions in psychiatric disorders. EEG mental arithmetic enables to assesse cognitive abilities in schizophrenic patients, by inducing brain activity that can be observed through frequency band analysis

**Objectives:**

This study aimed to assess the absolute spectral density (ASD) of various EEG frequency bands in schizophrenic patients and healthy controls, at rest and during a mental arithmetic task, in order to identify specific neural differences

**Methods:**

We conducted a cross-sectional, descriptive, and analytical case-control study involving 15 schizophrenic patients and 15 healthy controls. The study was carried out at the outpatient unit of Psychiatry Department “C” at Hedi Chaker University Hospital in Sfax, Tunisia. Participants underwent a standard wakefulness EEG with eyes closed at the Functional Explorations Department of Habib Bourguiba Hospital in Sfax, Tunisia. Each participant also performed a mental calculation test during the EEG recording

**Results:**

The ASD of the different EEGs was studied for each frequency band in the different cerebral lobes (Table 1). At rest, in schizophrenic patients, a significant difference was found between the mean ASD of the delta, theta, and beta1 frequency bands in the right and left occipital regions. Also, frequency band activity was more diminished in the left occipital regions than in the right. After mental calculation, the interhemispheric asymmetry disappeared in schizophrenic patients. In the control group, no significant differences were found between the mean ASD for any frequency band in the right and left frontal, temporal, and occipital regions, either at rest or after mental calculation (Table 1)

Table 1 : Comparison of EEG absolute spectral density between hemispheres in schizophrenics and controls at rest and after mental calculation task

**Table:** 

	*Schizophrenics*		*p=*	*Controls*		*p=*
	*Right*	*Left*		*Right*	*Left*	
**At rest**						
**DELTA** Occipital	30,44± 26,37	22,74±19,34	**0,021**	5127,7±19031	5111,41±18968	0,373
**THET** Occipital	58,17±47,67	45,67±44,88	**0,023**	3856,8±12450	3863,76±12458	0,721
**BETA 1 **Occipital	7,22±4,25	6,24±3,77	**0,002**	497,9±1854	498,26±1860	0,854
**After mental calculation task**						
**DELTA** Occipital	22,62±17,8	24,84±33,76	0,725	4970,14±15006	4979,39±15068	0,762
**THETA** Occipital	46,35±41,48	62,62±96,01	0,442	1895,46±5301	1849,43±5245	0,766
**BETA1** Occipital	5,76±3,45	5,43±3,17	0,466	934,81±3152	919,04±3093	0,328

**Conclusions:**

These results underline the importance of considering cerebral lateralization in the diagnostic and therapeutic approach to schizophrenia. It is important to note that mental arithmetic involves complex cognitive processing, essentially working memory. Hence the importance of adopting a therapeutic approach incorporating not only pharmacological treatments, but also cognitive remediation therapies

**Disclosure of Interest:**

None Declared

